# Serpentine supravenous hyperpigmentation: a rare clinical image

**DOI:** 10.11604/pamj.2024.47.189.43001

**Published:** 2024-04-16

**Authors:** Ashwin Karnan

**Affiliations:** 1 Department of Respiratory Medicine, Jawaharlal Nehru Medical College, Datta Meghe Institute of Higher Education and Research, Sawangi (Meghe), Wardha, Maharashtra, India

**Keywords:** Pigmentation, ulcer, carcinoma, chemotherapy, melanin

## Image in medicine

A 39-year-old male presented with complaints of left arm skin discoloration associated with itching for the past 2 weeks. The patient has a known case of signet ring cell carcinoma of the stomach for the past 6 months and has completed 6 cycles of intravenous 5-fluorouracil through a peripheral venous catheter. A biopsy of the skin showed melanophages with perivascular mononuclear cell infiltration. A diagnosis of 5-fluorouracil-induced hyperpigmentation was made. The patient was treated with topical steroid application and antihistamine and is currently on follow-up. Serpentine supravenous hyperpigmentation was described by Hrushesky in 1976 which is a cutaneous reaction to intravenous antineoplastic agents. It is seen in 2-5% of patients receiving intravenous chemotherapy drugs but is a benign and self-limiting disease. The chemotherapy agents include cyclophosphamide, actinomycin, bortezomib, doxorubicin, and 5-fluorouracil. The exact mechanism is unclear, but possible hypotheses include endothelial damage leading to extravasation of the drug and interference with melanogenesis, direct stimulation of melanocytes, hyperthermia-related increased expression of melanocyte-stimulating hormone receptor, and hyperpigmentation secondary to increased blood flow. The condition is reversible, usually months to years after cessation of the drug. It can be prevented by using a central chemo port for drug administration.

**Figure 1:**
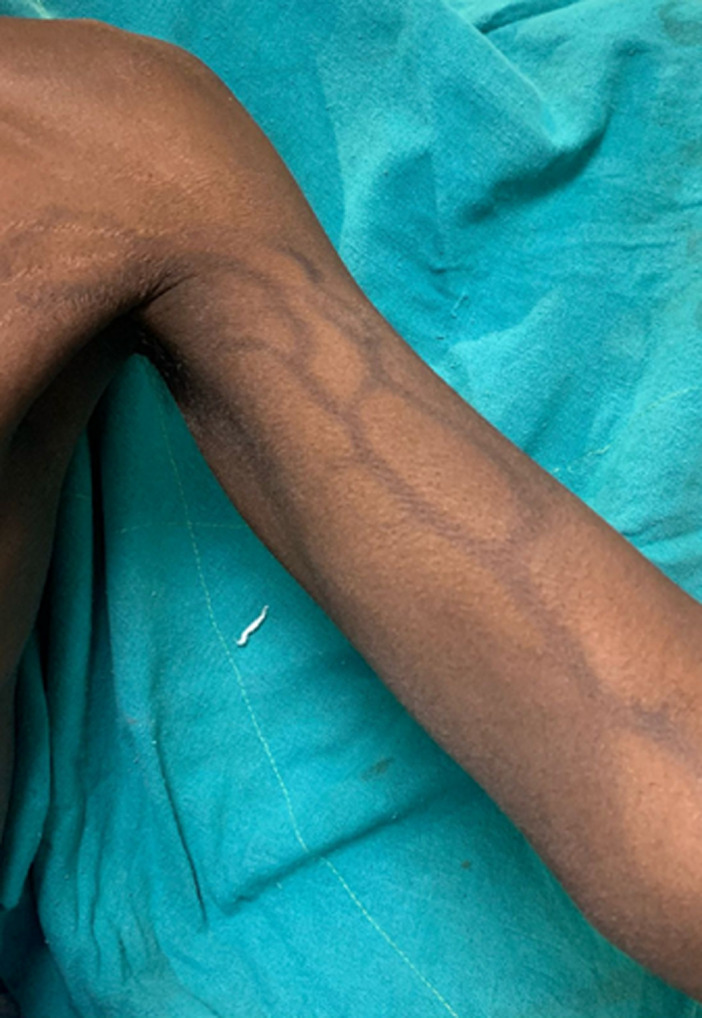
hyperpigmentation of the left upper arm veins

